# Bicuspid Aortic Valve Disease: From Pathophysiology to Treatment

**DOI:** 10.3390/jcm13174970

**Published:** 2024-08-23

**Authors:** Odysseas Katsaros, Nikolaos Ktenopoulos, Theofanis Korovesis, Georgios Benetos, Anastasios Apostolos, Leonidas Koliastasis, Marios Sagris, Nikias Milaras, George Latsios, Andreas Synetos, Maria Drakopoulou, Sotirios Tsalamandris, Antonios Karanasos, Konstantinos Tsioufis, Konstantinos Toutouzas

**Affiliations:** 1 First Department of Cardiology, National and Kapodistrian University of Athens, Hippokration General Hospital of Athens, 11527 Athens, Greece; faniskorovesis@gmail.com (T.K.); benetosg@gmail.com (G.B.); anastasisapostolos@gmail.com (A.A.); lkoliastasis@gmail.com (L.K.); masagris1919@gmail.com (M.S.); glatsios@gmail.com (G.L.); synetos@yahoo.com (A.S.); mdrakopoulou@hotmail.com (M.D.); stsalamandris@hotmail.com (S.T.); ktsioufis@gmail.com (K.T.); ktoutouz@gmail.com (K.T.); 2 Medical School, European University of Cyprus, Nicosia 1516, Cyprus; 3 Department of Cardiology, Patras University Hospital, 26504 Patras, Greece; akaranasos@hotmail.com

**Keywords:** bicuspid aortic valve, aortic valve disease, aortic dilatation, congenital heart disease, surgical aortic valve replacement (SAVR), transcatheter aortic valve replacement (TAVR), pathophysiology, hemodynamic effects, future directions, bicuspid aortic valve registry

## Abstract

The Bicuspid Aortic Valve (BAV) is the most common congenital anomaly in adults, with a global incidence of 1.3%. Despite being well documented, BAV presents significant clinical challenges due to its phenotypic heterogeneity, diverse clinical manifestations, and variable outcomes. Pathophysiologically, BAV differs from tricuspid valves in calcification patterns and hemodynamic effects, leading to increased shear stress and aortic root dilatation, while it is influenced by genetic and hemodynamic factors. This is why therapeutically, BAV presents challenges for both surgical and transcatheter interventions, with surgical approaches being traditionally preferred, especially when aortopathy is present. However, transcatheter aortic valve implantation (TAVI) has emerged as a viable option, with studies showing comparable outcomes to surgery in selected patients, while advancements in TAVI and a better understanding of BAV’s genetic and pathophysiological nuances are expanding treatment options. The choice between mechanical and bioprosthetic valves also presents considerations, particularly regarding long-term durability and the need for anticoagulation. Future research should focus on long-term registries and genetic studies to refine therapeutic strategies and improve patient outcomes. This review aims to evaluate current approaches in the surgical and interventional management of BAV, focusing on its anatomy, pathogenesis, pathophysiology, and therapeutic strategies.

## 1. Introduction

The Bicuspid Aortic Valve (BAV) is the most common congenital anomaly encountered in the adult population, with an incidence of 1.3% worldwide and is characterized by non-negligible complications and mortality [1,2]. From its first description by Leonardo Da Vinci more than 500 years ago and the groundbreaking description by Drs William Osler and Maude Abbot, our understanding of BAV has evolved significantly. Nonetheless, this well-described valvuloaortopathy comprising of multiple grades of valve dysfunction and aortic dilatation that develop during the patients’ lifetime continues to surprise medical professionals with its phenotypic heterogeneity, its variance in clinical manifestations and also its multiple correlations and outcomes. Therefore, it was all but inevitable for researchers to try and explore the less than clear aspects such as the genetics of BAV, the mechanisms of its dysfunction, and the clinical outcomes, in order to develop novel therapeutic strategies.

In most cases. BAV is isolated, not correlated with a clinical syndrome, and despite recent breakthroughs, its genetic background is not well understood. Despite multiple correlations and hypotheses, it is still not clear why some BAVs present with stenosis while others with insufficiency. The mechanism behind the rapid degeneration of some BAVs while others remain clinically silent and undiagnosed is still not elucidated. Furthermore, the reason why some BAV patients present with isolated aortovalvulopathy while others present with genetic syndromes or other congenital heart diseases is also unknown.

The purpose of this review is the concise evaluation of BAV with a focus on current approaches in surgical and interventional management.

## 2. Anatomy of Bicuspid Aortic Valve

Bicuspid Aortic Valve is defined by the presence of two functional commissures between the cusps with less than three zones of parallel placement in between. The presence and orientation of the raphe fusion presents high variability between individuals. The fuses may be congenital or acquired—due to a rheumatic disease or due to age-related atherosclerosis. Theoretically, there is a plethora of possible degrees and combinations of fuses between the cusps.

**Figure 1:**
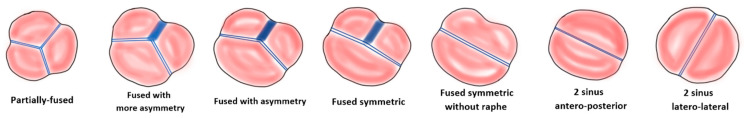
Expert consensus on classification of BAV.

**Figure 2:**
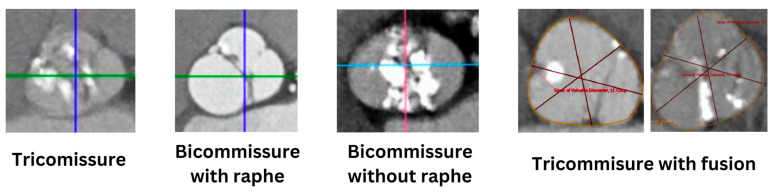
BAV classification in the TAVI era [7]. Images are derived from Computed Tomography scans. Blue lines at the first two images and pink line at the third image represent the vertical axis. Green lines at the first two images and light blue line at the third image represent the horizontal axis. The burgundy colored lines at the last two images were used to indicate the different dimensions during measurements.

Most BAV classifications in the published literature stem from surgical analyses. The fuse between the right and left coronary cusp is the most common type, occurring in nearly 80% of the cases, followed by the fusion of the right and non-coronary cusp, while more rarely, the left and non-coronary cusps are fused. The most widely used classification of BAV is the Sievers classification, which describes the number and orientation of the fuse based on surgical models [3]. Type zero contains no fusion with two functional symmetrical cusps, type 1 contains a fusion between two subdeveloped cusps, type two contains two fusions with two maldeveloped cusps and one normally developed cusp. There is a simplified classification system, based on the two fused cusps, which has been adopted by the International BAV Consortium (BAVCon) in 2014. All three types (type 1: fusion of left–right coronary cusp, type two: fusion of right non-coronary cusp, type three: fusion of left non-coronary cusp) may or may not contain raphe [4]. De Kerchove et al. proposed a classification system which evaluates the feasibility of surgical repair such as the orientation of the raphe, the length of the fusion, and the height of the commissure [5]. Recently, a new consensus statement has been developed regarding the nomenclature of BAV with a simple and complete classification system based on imaging modalities (Computed Tomography—CT, echocardiography, Cardiac Magnetic Resonance—CMR) and the anatomy of the surgical pathology (Figure 1) [4,6]. There are three types of BAV described in this consensus: the fused (which is similar to Sievers type 1), the two-sinus (referring to latero-lateral and antero-posterior phenotypes), as well as the partially fused types. There is then further subclassification of the fused type according to the symmetry of the functional cusps and the commissural angle. Jilaihawi et al. [7] have adapted the Sievers classification in order to tackle the interaction of the transcatheter valve with the aortic root. The BAV morphologies were defined as bicommisural without raphe (equivalent to Sievers type zero), bicommissural with raphe (equivalent to Sievers type 1), and tricomissural (which presents common features between Sievers type 1 and tricuspid valves) (Figure 2).

In a pilot study the 30-day mortality, stroke, and pacemaker implantation incidence was similar in all BAV morphologies [7]. It is interesting to note that the intercommisural distance in bicommisural bicuspid valves correlated with at least moderate paravalvular leak (PVL), despite the relatively low power of the study (n = 130). In contrast to the STS database which started collecting data on anatomical BAV features in 2017, the STS/ACC TVT registry does not contain info on the classification on the BAV subtype. The effect of BAV morphology in Transcatheter Aortic Valve Intervention (TAVI) outcomes is still scarcely explored. In an international multicenter BAV-TAVI registry, BAVs were classified according to a modified Sievers classification which differentiates the calcified raphe morphology from an uncalcified type 1 raphe. One-year mortality was significantly higher among type zero, type 1 with uncalcified raphe, and type 1 with calcified raphe (2.4%, 4.8%, and 9.5%, *p* = 0.006). Moreover, patients with calcified raphe and extended calcification in the cusps, had at least significantly higher moderate PVL (*p* = 0.002) and two-year mortality in comparison to patients with none of the above features (25.7% vs. 5.9%; log-rank *p* < 0.001) [8].

## 3. Pathogenesis and Genetic Determinants of Bicuspid Aortic Valve

There are multiple methods of inheritance described, such as autosomal dominant, X-linked, and familial modes. Mutations in NOTCH1 (which correlate with de-suppression and calcium deposition in BAV) and GATA5 (which correlate with aortopathy) have been identified [1,9,10,11,12]. Those abovementioned genes discovered in families with BAV—such as NOTCH1—have also been identified in sporadic cases of BAV, so they can pose a starting point for understanding the genetic architecture. The notable heterogeneity poses a challenge for researchers who try to identify genetic variations that lead to BAV or predict BAV-associated complications. To deal with these challenges, the following goals should be achieved: A well-defined BAV cohort should be created and closely followed up in the long term. It should be large enough to allow correlation studies adequately powered to identify genetic variants with small or moderate interactions leading to BAV and its related complications and examine genetic determinants of rare events or BAV subtypes. Numerous patients will be required in order to identify important genetic contributors. Secondly, bias due to misclassification should be minimized. All cases of a BAV registry should be thoroughly evaluated by experts in the field, using stern unified criteria and standardized measurements. The evaluation of the phenotype and of the clinical endpoints with the proper imaging core labs will deter misclassification and ensure the credibility of the findings. Also, follow-up should be long enough in order to record late BAV complications, such as stenosis, prioritizing the registry of individuals who have not yet presented with a disease. This requires follow-up of more than 10 years. The inclusion of retrospectively identified patients is of utmost importance, if precise phenotype estimations and standardized measurements in an available imaging method were applied. Furthermore, a negative control group should also be recruited with tricuspid aortic valves and be followed up parallel to the BAV cohort. To minimize confounding factors due to systemic differences between patients and controls, it is important to compare BAV group findings to appropriately matched controls stemming from the same populations as the BAV group. The correlations may be warped by confirmation bias if convenience control groups are used from registries or biobanks, which have not received the same level of diagnostic quality. Last but not least, families with a history of BAV should be prioritized, because of the increased chance to carry rare variations in solitary genes which can be identified using whole-exome sequencing or whole-genome sequencing.

**Figure 3:**
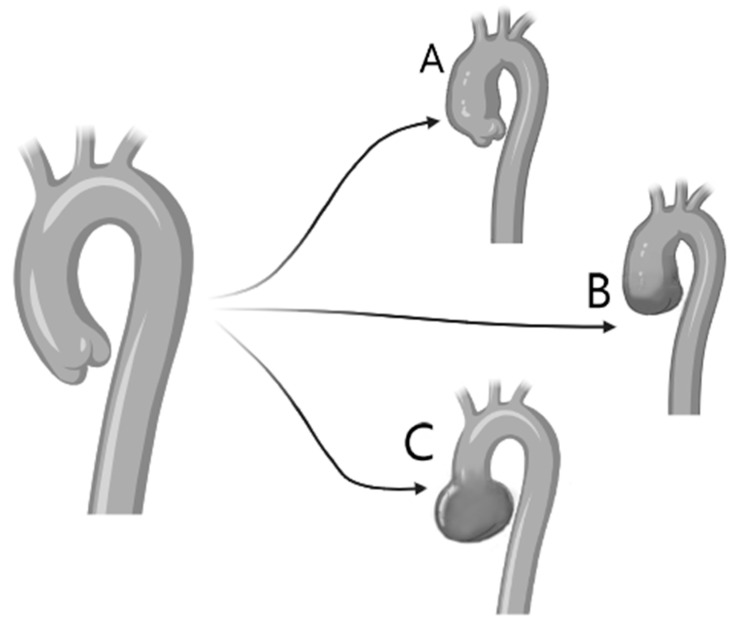
Schematic representation of the varying aortic disease phenotypes in BAV. The differing motifs of dilatation that can occur with BAV are compared to the normal aorta (top left). Although the most common distended segment is the tubular ascending aorta (**A**), the whole ascending aorta can be affected, including the sinuses of Valsalva and the tubular aorta with elimination of the sinotubular junction (**B**). A subgroup of patients can present with dilatation in the sinuses of Valsalva (**C**). This motif is correlated with type 1 BAV and male sex [4,13,14].

As genetic risk factors are identified, the registry should be the basis for clinical trials of genetic tests or therapies for BAV patients at high risk for adverse endpoints, in order to develop targeted medical therapies and up-to-date surgical decision making based on individualized genetic risk profiles (Figure 3) [4]. As multiple variations will likely contribute to BAV outcomes, we envision a multicenter registry as an opulent and continuous source of future genetic studies.

## 4. Pathophysiology

In comparison to tricuspid valves, BAV has different localization and increased calcification of the aortic valve [15]. The asymmetrical motion and higher commissural height increase the shear stress through the valve and lead to an accelerated calcification process starting from an early age. Another consequence of shear stress is the dilatation of the aortic root and ascending aorta.

Higher annulus and Valsalva sinuses dimensions have been reported in BAV patients undergoing TAVI (mean area—derived annular diameter 26.3 ± 3.0 vs. 23.2 ± 1.9 mm, *p* < 0.01 and Valsalva sinus 930.0 vs. 866.6 mm^2^, *p* = 0.005) [16]. Recent blood flow analysis CMR studies have confirmed the increased shear stress of the aortic wall caused by eccentric jets [4]. A small cohort study reported an increased aortic dilatation correlated with the angle of the jet [17]. It should be noted that another blood flow analysis study has proposed a different degree of severity of flow disruption according to BAV type; therefore, it remains in preliminary studies [18].

Congenital anomalous coronary arteries from the opposite coronary sinus (ACAOS) are more common in congenital BAV compared to tricuspid valves (7% vs. 3%, *p* = 0.001) which mainly affect the anomalous origin of the right coronary artery [19]. While the similar incidence of anomalous left main origin has been observed among BAV and tricuspid valves, the lack of a left main with separate ostia of left anterior descending and circumflex artery have been reported to be more common in BAV compared to tricuspid valves [20]. Moreover, higher coronary ostial height has been reported in BAV [21].

## 5. Aortic Root Dilatation and Aneurysm Formation

The reported incidence of aortic root dilatation in BAV patients is in the range of 20–84% [1,2,10]. Differences in features of the study populations evaluation methods and lower dilatation limits during aortic root measurements, as well as the heterogeneity of the disease itself, may affect the incidence of aortic root dilatation [22]. The normal aortic root diameter is affected by multiple factors including age, sex, body dimensions, genetic factors, the point of measurement, as well as the accuracy of measurement [23,24]. In an adult population, the aortic root diameter is larger in males than females by 1–3 mm, while studies in children have not shown differences in diameter based on sex when corrected for body surface area (BSA) [24]. Genetic factors as well as hemodynamic theory have been proposed as factors playing a role in the development of valvulopathy and aortopathy in BAV and also intense exercise is believed to negatively impact the hemodynamic parameters and accelerate the dilatation of the aorta, making athletes with BAV at higher risk for dissection [25,26].

The rates of dilatation vary and are usually unpredictable in BAV patients, while the underlying mechanisms responsible for the different valvuloaortic phenotypes remain unclear [27,28]. An alternative route of BAV and aortopathy is the anomalous migration of cells from the neural crest [2,10]. Aortic root dilatation may be connected by histologic changes, previously named as cystic necrosis of the tunica media, as a result from aberrant regulatory pathways of the vascular smooth muscle cells within the tunica media. The abnormal processing of the protein of the extracellular matrix (ECM) fibrillin-1 from the smooth muscle cells kicks off the detachment of the smooth muscle fibers from the ECM leading to the release of matrix metalloproteases (MMPs) together with their tissue inhibitors [22]. The resulting matrix degeneration and lamellar fragmentation leads to the increased apoptosis of smooth muscle fibers and disruption of the tunica media, negatively impacting the structural integrity and elasticity of the aorta [1,2,10].

Hemodynamic factors may also negatively impact the aortic root dilatation: the presence of abnormal valve dynamics due to the morphology of BAV may lead to aortopathy. Even normally functioning BAVs may present abnormal motifs of transvalvular flow resulting in increases in shear stress, which can be predicted to a degree by the morphological features of the BAV [9,29].

**Figure 4:**
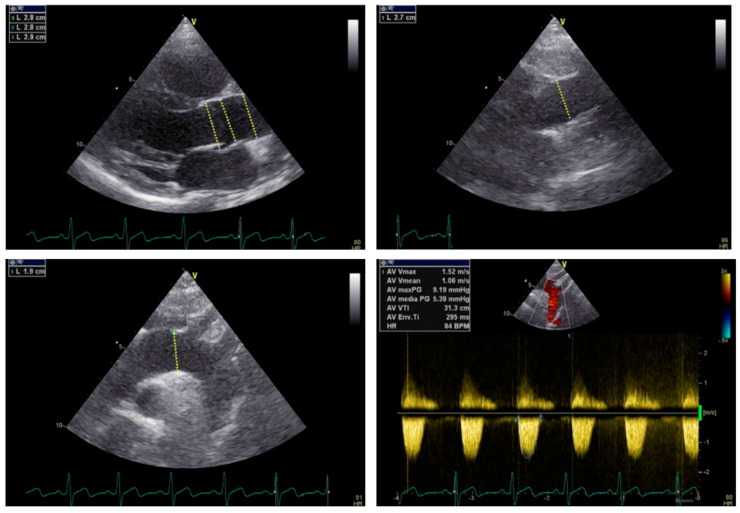
Echo of aortic dilatation in a 14-year-old patient with BAV. Evaluation of the aortic arch, including that the transaortic pressure gradient in the descending aorta should be necessary in all athletes and especially BAV athletes.

The type of BAV also impacts the dilatation of the aorta: type 1 includes a dilatation of the tubular ascending aorta (especially along the length of its curvature), accompanied by varying degrees of aortic root dilatation. This type has been associated with older age at diagnosis (>50 years) and valve stenosis. Type 2 includes isolated disease of the tubular ascending aorta which often extends to the transverse aortic arch. Type 3, also called root phenotype, includes isolated dilatation of the aortic root. This rare type correlates with younger age at diagnosis (<40 years), male sex, and aortic insufficiency while it is proposed that it is a form of bicuspid aortopathy which is likely to be connected to genetic causes [9,10,13,30,31]. Therefore, it is recommended that patients with BAV undergo full echocardiographic evaluation of the aortic valve, where the BAV motif and the function of the aortic valve and the aorta will be determined to avoid missing the diagnosis of aortic root dilatation (Figure 4). Novel data further demonstrate the atypical pattern of dilatation which is more common in BAV and poses a challenge in estimating the true diameter [32]. The impact of BAV in aortic disease is highlighted in the 2022 ACC guidelines on aortic disease which support that a 0.3 cm increase in diameter should be considered a rapid ascending-aortic aneurysm growth, which is an indication for intervention [33,34].

Age also has an impact in aortic root dilatation—especially in athletes; all the segments of the ascending aorta are usually larger in adults with BAV compared to those with tricuspid aortic valve [22]. Aortic dilatation usually starts from childhood and is progressive, with the incidence of dilation at the level of the tubular ascending aorta increasing with age [35].

## 6. Treatment

As mentioned, BAV can present with aortic regurgitation, aortic stenosis, or a combination of both. This distinction is critical in determining the appropriate treatment strategy, as the presence of regurgitation, stenosis, or both influences the decision between proceeding with TAVI or surgical intervention.

### 6.1. Challenges in Transcatheter Interventions

The BAV anatomy may be classified in multiple phenotypes according to the number of fusions, with type 1 consisting of a large central raphe, which comprises 90% of the affected individuals, without these phenotypes having a prognostic significance in TAVI outcomes [3,36]. According to a study by Yoon et al., which included 927 patients with type 1 BAV and a high grade of calcification, patients had higher rates of PVL and mortality at 30 days and 2 years, which underlines the role of calcium and valve morphology in selecting the proper patients for each treatment modality [8].

Traditionally, the surgical approach for BAV is preferred as the main treatment of choice [37,38]. In contrast to the degenerative disease of a tricuspid valve, BAV disease is a result of a genetic defect which affects the development of the heart and great vessels. In almost 50% of patients, there is a defect not related to the valve, most commonly as a result of the aortopathy presenting in 20–40%, while factors such as tunica media degeneration pose a risk factor for aneurysm as well as dissection of the ascending aorta. In patients with BAV stenosis with an indication for replacement, elective replacement of the ascending aorta is recommended when its diameter is increased to more than 45 mm [37]. Therefore, if another pathology coexists, surgery is the treatment of choice. If, however, there is only valvulopathy, there are still some challenges which should be considered for the choice of proper therapy [38].

Initially, aortic valve dimensions and other morphological features such as the aortic annulus are larger in BAVs, which increases the chance that the size of the transcatheter valve needed may be outside of the spectrum of the commercially available TAVI valves [38]. Secondly, the aortic annulus has an elliptic shape in BAV which poses a problem for the available devices which are designed for a round annulus [39]. The use of these devices comes with an increased risk of suboptimal apposition, resulting in PVL. Thirdly, BAVs tend to be more heavily and asymmetrically calcified, which leads to more troubles with implantation, with the ever-present risk of PVL [40].

### 6.2. Transcatheter versus Surgical Replacement

A study by Nagaraja et al. regarding TAVI in BAV patients which used data from the US Ministry of Health showed that between 2011 and 2014, only 1% of the patients who underwent TAVI had BAV. Despite the fact that these are younger patients with less comorbidities, they had similar rates of hospital mortality and serious complications compared to those with tricuspid valve [40]. Later, an updated publication of the previous study for the years 2012–2016 reported that BAV patients comprised 3.3% of the total TAVI patients, while comparison of transcatheter to surgical intervention revealed similar in-hospital mortality [41]. In another study by Sanaiha et al., data from 68% of the hospitalizations in the US were used [42]; 623,721 patients with aortic stenosis who underwent replacement of aortic valve were studied, of whom 563,331 [9], had BAV. Overall, TAVI was used in 6.8% of the procedures for BAV stenosis. This number increased 21 times during the study period, while in 2019, at least 20% of the patients who underwent TAVI had BAV. The number of centers that performed TAVI in BAV increased from 25 to 221. Concurrently, it comes as no surprise that patients who underwent TAVI were of older age and with more comorbidities in comparison to those subjected to surgical replacement [41,42,43]. In this analysis, the primary endpoint of mortality during hospitalization was higher for TAVI patients (1.6% vs. 0.8%), while major complications (mainly bleeding) were higher in the surgery group (33.6% vs. 22.2%). These differences were not observed after adjusting for risk. The rates of all-cause readmission were higher in the 90-day follow-up period for the TAVI group, a difference that was also eliminated after risk adjustment. Irrespective of statistical analysis, no major differences in 30-day readmission were observed. Although these factors contribute to the increase in TAVI cost, the hospitalization cost was also higher for TAVI despite the shorter duration of stay, which only serves to underline the impact of its cost [38].

Furthermore, the data from Sanaiha et al. [42] showed that TAVI in BAV correlated with higher rates of reoperation in comparison to surgery and also in comparison to TAVI in patients with tricuspid valve (5.9% vs. 4.1%). It seems that previous long-term considerations for the TAVI therapy in BAV seem to fade away as the method becomes more popular. This seems to be desired, as certain studies prove that the risk of in-hospital morbidity and mortality are comparable between the two approaches in this population. This is supported by the US FDA decision to remove the warning about BAVs from the transcatheter valves. Of course, careful patient selection is required [44]. Not all patients with BAV are similar and we must learn to identify the best candidates more accurately for each approach. Finally, experience from other practices should be considered such as surgery after TAVI, Valve-in-Valve TAVI, or even Valve-in-Valve-in-Valve TAVI [45]. Similar to how TAVI became a common therapeutic choice in patients with BAV when it was previously contraindicated, these scenarios may well take place in the near future and should be followed up closely.

### 6.3. Comparison between Mechanical and Bioprosthetic Valves

Aortic valve replacement with mechanical or bioprosthetic valves is the method of choice for the treatment of BAV stenosis [46]. Although bioprosthetic valves are effective in improving the hemodynamics of the disease, there are certain long-term disadvantages which become more pronounced when taking younger patients into account, which are practically the majority of patients with BAV [46]. Mechanical valves are more resistant [47] but have higher rates of reoperation which vary from 0.5% to 1% per patient-year [48]. Moreover, it seems that survival in non-elderly individuals after aortic valve replacement with a mechanical valve is significantly lower than in the general population, who present with 1% mortality per year [49]. The need for continuous antithrombotic therapy has an impact on the patients’ quality of life and is connected with a higher risk of serious thrombotic and bleeding events [50,51].

On the other hand, patients receiving bioprosthetic valves incur a higher risk of valve degeneration, thromboembolism, patient-prosthesis mismatch (PPM), and need for reoperation [52]. Despite the increased interest in bioprosthetic valves, especially during the era of Valve-in-Valve TAVI, many studies demonstrate superior results for mechanical compared to bioprosthetic valves [53,54]. Lately, there has been a renewed interest in Ross operation in adults, with good long-term survival, and low complication rates. Of course, it should be noted that this operation is technically complex, with a high risk of reoperation, especially in patients with aortic insufficiency. These rates improve in high-volume experienced centers [55].

### 6.4. Suggested Approach to Therapy Selection

BAV patients are often excluded from large, randomized trials, despite the fact that 5–10% of patients undergoing TAVI have BAV [56]. The main concerns associated with BAV stenosis are usually its anatomic features, mainly the presence of a calcified raphe and its size, or the occurrence of eccentric calcification and concomitant aortopathy [57].

Aortic root dilatation at the level of Valsalva sinus or the proximal segment of the ascending aorta coexists in 20–80% of BAV adults while some develop aneurysmal dilatation (>45 mm) with increased risk of dissection or rupture [44]. In these patients’ replacement of the ascending aorta, it is necessary to avoid adverse events [57]. Surgical replacement of the aortic valve with or without concurrent replacement of the aortic root in BAV stenosis patients seems to carry low in-hospital mortality from 0,7% to 2.4% and high rates of 10-year survival, upwards of 80% [58]. Although the safety and efficacy of TAVI compared to surgery in patients with BAV stenosis is not determined through large, randomized studies, there are a number of observational studies with a large number of BAV patients that underwent TAVI and report comparable results to surgically corrected BAVs and tricuspid valves [56,59,60].

Based on the analysis of the STS/ACC TVT registry, it occurred that TAVI in BAV patients resulted in comparable hemodynamic outcomes with a slightly higher rate of moderate or severe PVL compared to TAVI in the tricuspid valve even with the use of modern devices [59,60]. A different analysis of the same record containing patients of low surgical risk who received SAPIEN 3/Ultra transcatheter valve did not show a statistically significant difference in stroke occurrence and mortality at 30 days and 1 year [60]. Finally, an analysis of the National Inpatient Sample database comparing surgical to transcatheter approaches in BAV showed significant differences, with the transcatheter approach being associated with lower rates of in-hospital complications, including mortality and stroke, compared to the surgical approach [41].

**Figure 5:**
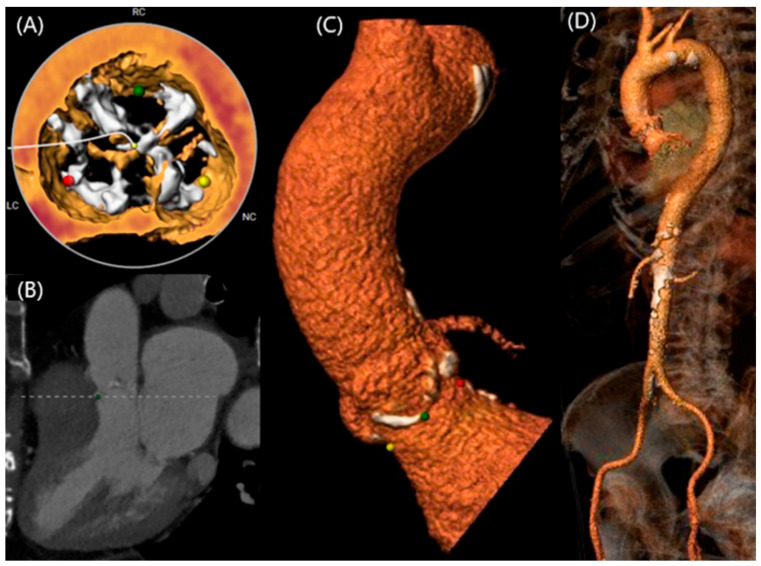
Female 93-year-old patient with severe BAV stenosis who underwent TAVI. Case from the Cath lab from “Hippokration” General Hospital Athens. Images from MDCT during preoperative planning demonstrating: (**A**) anatomy of the BAV and its degree of severity, (**B**) double oblique projection with CT reconstruction for the evaluation of the aortic annulus and ascending aorta, (**C**) 3D reconstruction of the aortic root and ascending aorta, (**D**) 3D reconstruction of the aortic root ascending aorta, aortic arch, descending aorta, and iliac arteries. The three color dots at the figure represent the 3D points for the visualization of the annular base.

The main morphological restrictions connected to BAV stenosis concern mainly its anatomic features, mainly the presence of a calcified raphe and its extend, the occurrence of calcifications and concomitant aortopathy, where predilatation may have a significant role in TAVI [61,62]. The proper identification of these features seems to be important in individualized decision making between TAVI and surgery for each patient. TAVI with modern devices seems to be safe and efficacious in elderly patients with BAV stenosis (Figure 5). However, the surgical approach should remain the main therapeutic choice for BAV stenosis in younger patients, especially in hostile anatomies or concomitant significant aortopathy [36,57].

## 7. Future Directions

The future of BAV management lies in a multidisciplinary approach that integrates genetic research, advanced imaging, dedicated devices, improved surgical techniques, AI-driven simulations, and novel therapeutic strategies.

Firstly, future research should focus on the genetic underpinnings of BAV and its related complications. A large-scale, longitudinal registry encompassing a well-defined BAV cohort is crucial for identifying genetic variants that contribute to BAV and its associated aortopathy. This registry should collect comprehensive data to enable the identification of genetic markers associated with disease progression and outcomes; standardize measurements to implement strict, unified criteria, and standardized imaging protocols to minimize misclassification and ensure the accuracy of data; follow patients over an extended period to capture late-onset complications and natural disease progression; and, utilize well-matched control groups with tricuspid aortic valves to identify genetic differences and control for confounding variables.

Afterwards, understanding the molecular and cellular mechanisms underlying BAV pathogenesis is essential and future studies should investigate how altered hemodynamic forces due to BAV lead to cellular and molecular changes in the valve and aortic wall that includes studying the role of mechanosensitive pathways in valvular calcification and aortic dilatation. Also, understanding of the inflammatory milieu in BAV patients could reveal new anti-inflammatory strategies for treatment.

Furthermore, advanced imaging modalities, such as 4D-flow MRI, should be developed and validated in order to provide detailed hemodynamic information and help predict disease progression; as well, non-invasive biomarkers should be identified and validated that can correlate with disease severity and progression, aiding in early diagnosis and monitoring of the response to treatment [63,64].

Additionally, advances in device technology and surgical methods are critical for enhancing the management of BAV and its complications. The design and development of specialized devices tailored specifically for BAV patients should address the unique anatomical and functional challenges posed by BAV and its associated aortopathy, while innovative and refined surgical techniques can reduce operative risks and recovery times and, in that way, improve the outcomes of BAV-related procedures.

To add to this, artificial intelligence (AI) and advanced simulation technologies hold significant promise in revolutionizing the treatment of BAV. Utilizing AI algorithms to analyze imaging data and predict disease progression is going to enhance early diagnosis and personalized treatment plans. Likewise, developing AI-based simulations to model the behavior of implantable valves in BAV patients can lead to the prediction of long-term performance, optimization of valve design, and valve selection personalization based on individual patient anatomy and hemodynamics.

As our understanding of BAV improves, novel therapeutic strategies need to be developed and tested, such are targeted medical therapies based on genetic and molecular insights, to prevent or slow down the progression of BAV-related complications. Also, advances in tissue engineering and regenerative medicine could lead to the development of biological valve replacements that mimic the natural valve’s biomechanical properties. Last but not least, exploring pharmacological agents that can modulate the shear stress-induced pathways, can thereby potentially delay the progression of valvular calcification and aortic dilatation.

## 8. Conclusions

BAV disease is a common genetic abnormality of the aortic valve, with Sievers type 1 being the most prevalent phenotype [65]. The diagnosis of BAV stenosis is increasing, partly due to the widespread use of Multi-Slice Computed Tomography (MSCT) in preoperative planning [66]. Recent studies have demonstrated that newer-generation artificial valves offer improved outcomes compared to earlier models, leading to the US FDA’s approval of self-expanding and balloon-expandable valves across all surgical risk categories, regardless of anatomy [67,68,69,70,71]. However, the unique morphology of the aortic valve in BAV patients increases the risk of complications such as PPM, PVL, thromboembolism, and annulus rupture [65].

Given that BAV is often diagnosed in younger patients, the use of TAVI requires careful interdisciplinary planning, particularly due to the association between BAV and aortopathy [72]. Although reliable data on disease progression in TAVI-treated BAV patients are limited, surgical data suggest that correcting valve hemodynamics may slow aortopathy progression [72]. A study by Jung et al. found no progression of aortic disease in BAV patients who underwent TAVI with aortic diameters < 50 mm over a mean follow-up of 398 days [73]. The potential for aortic injury during TAVI emphasizes the need for careful device selection and procedural techniques [62,74].

While short- and mid-term results for transcatheter valve longevity in BAV patients are encouraging, data on long-term outcomes remain limited [47,59,69]. Given the anatomical challenges posed by BAV, especially in younger patients with lower surgical risk and longer life expectancy, large, randomized controlled trials are essential to evaluate the long-term efficacy of transcatheter valves compared to surgical replacement [65]. Advances in understanding the genetics and biology of BAV stenosis, along with targeted therapeutic strategies, promise a future of personalized medicine, with improved treatment options for BAV-associated morbidity, including surgical and transcatheter interventions [65].
